# Association between Antihypertensive Therapy and Risk of Acute Lower Respiratory Infections (ALRI): A Retrospective Cohort Study

**DOI:** 10.3390/healthcare10112318

**Published:** 2022-11-18

**Authors:** Jana Heidemann, Marcel Konrad, Christoph Roderburg, Sven H. Loosen, Karel Kostev

**Affiliations:** 1 Epidemiology, IQVIA, 60549 Frankfurt, Germany; 2 Health & Social, FOM University of Applied Sciences for Economics and Management, 60486 Frankfurt, Germany; 3 Clinic for Gastroenterology, Hepatology and Infectious Diseases, Medical Faculty of Heinrich Heine University Düsseldorf, University Hospital Düsseldorf, 40225 Düsseldorf, Germany; 4 University Hospital Marburg, Philipps-University Marburg, 35037 Marburg, Germany

**Keywords:** antihypertensive therapy, acute respiratory infection, pneumonia, bronchitis

## Abstract

Purpose: The aim of this study was to analyze the association between antihypertensive drugs and the incidence of acute lower respiratory infections in patients treated in general practices in Germany. Methods: After propensity score matching of five antihypertensive drug classes, a total of 377,470 patients aged ≥18 years were available for analysis. The association between each antihypertensive drug class and ALRI incidence as compared to all other antihypertensive drug classes (as a group) was studied using conditional Cox regression analyses. Because of multiple comparisons and large patient samples, findings were clinically considered relevant when the hazard ratio was <0.85 or >1.15. Results: The regression analyses applied found no clinically relevant associations between antihypertensive drugs and the incidence of acute lower respiratory infections, as all hazard ratios were between 0.85 and 1.15. Conclusion: In the present study, only slight and not clinically relevant increases or decreases in the ALRI incidence were observed. Additional studies are necessary to further explore the risks associated with antihypertensive agents that are widely embedded in today’s clinical practice.

## 1. Introduction

Affecting approximately 1.4 billion people in 2010 and with a drastic increase to over 1.6 billion people expected by 2025 [1], hypertension remains the leading cause of cardiovascular disease and premature death worldwide [2]. Despite the growing incidence of hypertension, especially in low- and middle-income countries (LMIC), the global mean blood pressure (BP) has remained constant, which is mainly attributed to the widespread application of antihypertensive medication [2,3].

Today, a variety of antihypertensive medications, including ACE inhibitors ACEi, angiotensin II receptor blockers (ARBs), beta blockers (BB), diuretics, and calcium channel blockers (CCB), are used to prevent, control, and treat hypertension with the aim of reducing cardiovascular risks and obviating complications in patients suffering from heart failure, coronary heart disease, stroke, or diabetes [4,5].

With a consistent increase in the use of antihypertensive medication, much progress has been made in maintaining a stable systolic blood pressure (SBP) in hypertensive patients over the past few decades [6]. In this regard, a significant decline in the incidence of ischemic stroke has been achieved in recent years by improving risk factor control through stabilizing SBP [7].

However, despite the widespread application of antihypertensive therapy and a clear awareness of its impact in reducing the risk of cardiovascular morbidity, considerable controversy has developed among researchers about the potential association between antihypertensive drug use and the manifestation of acute lower respiratory infections (ALRI). Defined as an acute infection of the lower respiratory tract, including pneumonia and bronchitis [8], acute lower respiratory infections (ALRI) are considered a major threat to global public health and are co-responsible for the highest burden of disease measured by years lost through death or disability [9].

In this context, the possible protective effect of antihypertensives on ALRI risk has been at the center of discussions in recent years: In 1996, Sekizawa et al., found a reduction of about a third in the risk of pneumonia manifestation in patients treated with ACE inhibitors (ACEi) and thus emphasized the potentially beneficial effects of the drugs on the prevention of pneumonia [10]. These findings were confirmed by Van de Garde and colleagues in 2007, who found that diabetic patients were at a significantly lower risk of pneumonia when treated with ACEi [11]. Angiotensin II receptor blockers (ARBs) are also purported to lower the risk of (severe) pneumonia and mortality further than ACEi [12]. By contrast, a higher risk of pneumonia was observed in a case-control study conducted by Mukamal et al. in middle-aged Americans with beta blocker (BB), calcium channel blocker (CCB), and lipophilic ACEi use [13]. These findings are supported in other studies, refuting any protective effect induced by ACEi, BB, and CCB and even indicating an increased risk of ALRI and other infections in patients treated with antihypertensives [14,15].

In this context, the following study aims to evaluate the potential association between the intake of antihypertensive drugs and ALRI to supplement the insufficient evidence concerning the risks accompanying widely used antihypertensive therapy approaches.

## 2. Methods

### 2.1. Database

This study was based on data from the Disease Analyzer database (IQVIA), which contains drug prescriptions, diagnoses, and basic medical and demographic data obtained directly and in anonymous format from computer systems used in the practices of general practitioners and specialists [16]. The database covers approximately 3% of all outpatient practices in Germany. Diagnoses (according to the International Classification of Diseases, 10th revision [ICD-10]), prescriptions (according to the Anatomical Therapeutic Chemical (ATC) classification system), and the quality of reported data are monitored by IQVIA. In Germany, the sampling methods used to select physicians’ practices are appropriate for obtaining a representative database of general and specialized practices. It has previously been shown that the panel of practices included in the Disease Analyzer database is representative of general and specialized practices in Germany [16]. Finally, this database has already been used in previous studies focusing on antihypertensive therapy [17,18] as well as respiratory infections [19,20].

### 2.2. Study Population

**Figure 1:**
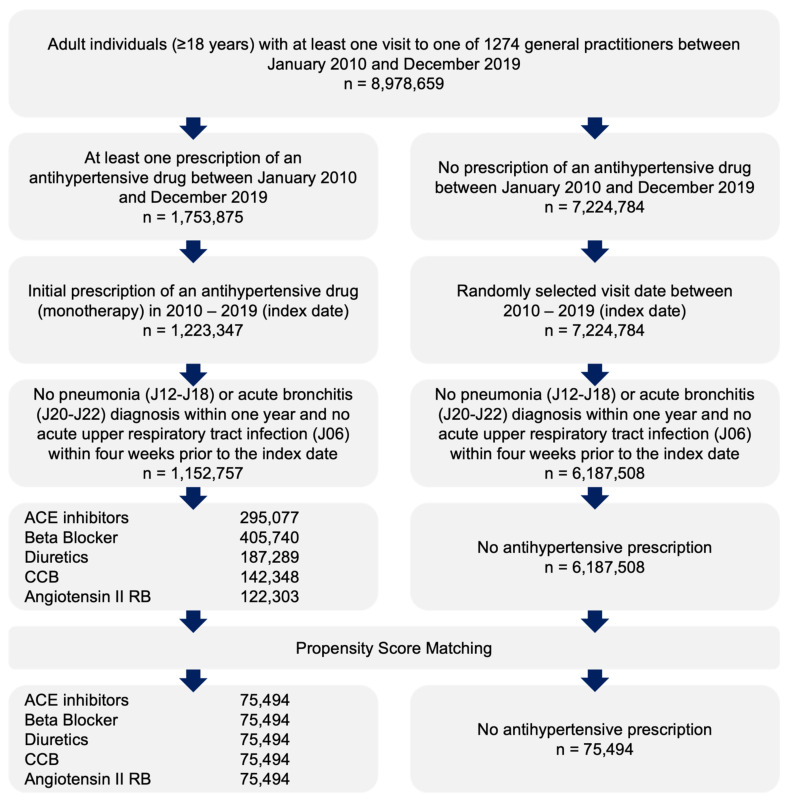
Selection of study patients.

This retrospective cohort study included adult patients (≥18 years) with an initial prescription of antihypertensive therapy alone (diuretics, ATC: C03A; beta blockers, ATC: C07A; calcium channel blockers, ATC: C08A; ACE inhibitors, ATC: C09A; angiotensin II receptor blockers, ATC: C09A) in 1274 general practices in Germany between January 2010 and December 2019 (index date; Figure 1). Patients diagnosed with pneumonia (ICD-10: C12–C18) or acute bronchitis (ICD-10: J20–J22) within 12 months prior to the index date or acute upper respiratory infection (ICD-10: J06) four weeks prior to the index date were excluded.

Patients receiving any of five antihypertensive drug classes were matched 1:1:1:1:1 to each other by propensity scores based on sex, age, and diagnoses documented within 12 months prior to or on the index date, including diabetes (ICD-10: E10–E11), ischemic heart diseases (ICD-10: I20–I25), heart failure (ICD-10: I50), renal failure (ICD-10: N18. N19), chronic bronchitis or obstructive lung disease (COPD) (ICD-10: J42–J44), and asthma (ICD-10: J45). Finally, patients without antihypertensive therapy were matched to one of five therapy cohorts with similar exclusion criteria (Figure 1).

### 2.3. Study Outcomes and Covariates

The main outcome of the study was the incidence of acute lower respiratory infection including pneumonia and bronchitis within 12 months after the index date as a function of antihypertensive therapy. Each patient was followed for up to 12 months from the index date until the first ALRI diagnosis was documented or until antihypertensive therapy ended (either because of a switch to another antihypertensive therapy or the addition of another drug class to the initial therapy). Patients without antihypertensive therapy were followed up for up to 12 months from the index date until the first ALRI diagnosis was documented or until the last visit date to the general practitioner.

### 2.4. Statistical Analyses

Differences in the sample characteristics between six cohorts (five cohorts with different antihypertensive drug classes and one cohort without therapy) were measured. Conditional Cox regression models were applied to study the association between each antihypertensive drug class and ALRI incidence compared to all other antihypertensive drug classes (as a group). These models were applied separately for four age groups and for women and men. As sensitivity analysis, the outcome was also defined as the diagnosis of ALRI plus a prescription of an antibiotic drug (ATC: J01) within seven days following the ALRI diagnosis. Both regression models were adjusted for the respective physician’s practice to reflect the diagnosis behavior of treating physicians. In addition, each antihypertensive drug class was compared to the cohort without therapy. To counteract the problem of multiple comparisons and also due to the large patient samples, *p*-values <0.001 were considered statistically significant. Because of multiple comparisons and large patient samples, findings were clinically considered relevant when the hazard ratio was <0.85 or >1.15. Analyses were carried out using SAS version 9.4 (SAS institute, Cary, NC, USA).

### 2.5. Ethical Statement

The database used includes only anonymized data in compliance with the regulations of the applicable data protection laws. German law allows the use of anonymous electronic medical records for research purposes under certain conditions. According to this legislation, it is not necessary to obtain informed consent from patients or approval from a medical ethics committee for this type of observational study that contains no directly identifiable data.

Because patients were only queried as aggregates and no protected health information was available for queries, no Institutional Review Board approval was required for the use of this database or the completion of this study.

## 3. Results

### 3.1. Basic Characteristics of the Study Sample

The present study included 75,494 patients being treated with each therapy class as well as those in the cohort without therapy (452,694 patients in total). The basic characteristics of the study patients are displayed in Table 1. Due to the matched pair design of the study, the age, sex, and comorbidity structure was the same for all five cohorts. The mean age (SD) was 65.3 (SD: 14.0) years, 56.9% were women, the prevalence of diabetes was 13.2%, that of ischemic heart diseases 6.6%, heart failure 1.7%, renal failure 2.0%, COPD 3.7%, and asthma 2.4%.

### 3.2. Cumulative Incidence of ALRI Diagnoses

**Figure 2:**
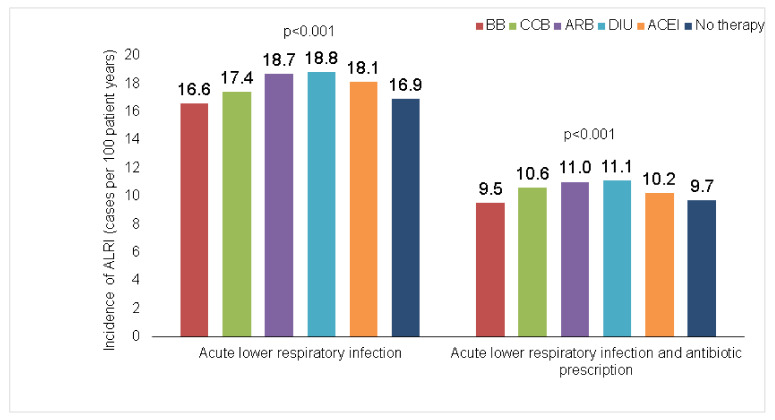
Proportion of individuals with a diagnosis of lower respiratory infection and antibiotic therapy within 12 months in patients treated with different antihypertensive drugs.

Figure 2 shows the incidence (cases per 100 patient years) of ALRI diagnoses. This incidence was slightly higher in patients treated with diuretics (18.8%), followed by ARB (18.7%) and ACEI (18.1%). The lowest incidence was in patients treated with BB. The same order was observed in terms of antibiotic therapy.

### 3.3. Association between Antihypertensive Therapy and Incidence of ALRI

Table 2 shows the results of the conditional regression analyses. The regression analyses applied found no clinically relevant associations between antihypertensive drugs and the incidence of acute lower respiratory infections, as all hazard ratios were between 0.85 and 1.15. For example, ARB therapy was associated with a slightly increased incidence of ALRI (HR: 1.08, 95% CI: 1.05–1.12) as well as antibiotic prescription (HR: 1.09; 95% CI: 1.04–1.13) compared to other antihypertensive drug classes. However, this association was only confirmed in male individuals and individuals aged 71–80. In the youngest age group (≤60 years), a positive association with ALRI was observed for individuals treated with ACEI.

Although CCB therapy was associated with a slightly reduced ALRI incidence (HR: 0.93; 95% CI: 0.90–0.96), this association was only observed in women (HR: 0.91, 95% CI: 0.87–0.95) and was not confirmed for antibiotic therapy.

Compared to the cohort without therapy, ARB also was associated with a slightly increased incidence of ALRI (HR: 1.10, 95% CI: 1.05–1.15) as well as antibiotic prescription (HR: 1.12; 95% CI: 1.05–1.19) (Table 3).

## 4. Discussion

This retrospective cohort study with a sample size of 452,694 patients under hypertensive therapy (75,494 patients in each group) generally identified no clinically relevant associations between antihypertensive drugs and the incidence of acute lower respiratory infections.

All antihypertensive agents investigated in this study have been examined thoroughly in previous studies and remain widely embedded in today’s clinical practice, however, the current evidence does not provide a consensus on possible associations between these agents and ALRI manifestation. Contrary to the findings of this study, ARBs and ACEi have recently been discussed as playing a protective role in the development of ALRI. While silent aspiration remains a major risk factor for the development of pneumonia [21], it has been hypothesized that the intake of ACEi might reduce the risk of aspiration by inducing a cough reflex through blocking degradation of substance P and bradykinin [22]. These two inflammatory peptides play a major role in sensitizing the sensory nerves of the airway, and thus, through inhibited metabolism, increase the cough reflex [23]. In congruence with these findings, Lin et al. also added inhibited virus invasion, modulation of the cholinergic pathway in the respiratory tract, and modulations of inflammation in lung parenchyma as beneficial attributes of the intake of ACEi [24]. These findings have been confirmed by other researchers, such as Caldera et al. [25] and Liu et al. [26].

However, studies that have found ACEi to be significantly beneficial to the development of ALRI have mainly been conducted in Asia or among populations in specialized clinics, such as post-stroke patients, which may have distorted the findings and their applicability to the general population. A Dutch case–control study aiming to shift the focus from previously studied subgroups towards the general population conducted by van de Garde and colleagues could not confirm any positive influence of ACEi on pneumonia risk among a general, largely white population [23]. Ethnic differences as found by Ohkubo et al., who identified an OR of 0.53 (95% CI, 0.33–0.86) in Asian ACEi users versus an OR of 0.95 (95% CI, 0.71–1.27) in non-Asian ACEi users, further strengthen this hypothesis [27,28]. The design of this German-based study with a broad population of 75,494 patients under ACEi therapy could therefore be a reason for the contradiction in findings compared to studies involving only individual subpopulations. However, the question of whether differences in genetic background between Asian and non-Asian populations may influence the effect of ACEi on ALRI manifestation remains open to speculation. Given the orientation of previous studies towards subpopulations, it is possible that the beneficial effects of ACEi with respect to ALRI development are pronounced in these targeted groups but might differ within a therapeutic setting of younger and especially white patients. By way of example, in a nested case–control study, Mukamal et al. identified lipophilic ACEi as being associated with a higher risk of pneumonia in middle-aged Americans with hypertension [13]. A possible explanation for the slightly increased risk of developing ALRI under ACEi therapy might evolve from a study conducted in 2021: Cao et al. demonstrated a significant inhibition of bactericidal activity in human and murine neutrophils when exposed to ACEi, which could induce higher susceptibility to bacterial infections [29]. However, future studies are essential to confirm these findings in a general population and thus gain an overall impression of the effects on ALRI risks accompanying the use of ACEi.

In addition to ACEi, the second most commonly used RAS-blocking agent—ARB—has been studied and discussed intensively among researchers in terms of its possible beneficial effect on ALRI manifestation. In previous examinations, anti-inflammatory effects as well as the alleviation of lung injuries have been described as favorable attributes of ARBs [30]. Again, relevant studies have relied mainly on specific subpopulations including Asian, neurologically impaired, and elderly patients [30]. Although the evidence concerning the protective effects of ARBs with respect to ALRI remains contradictory [21,27], there seems to be a common understanding among most researchers that ARBs are not associated with an increased risk of ALRI. By way of example, in their case-crossover study, Liu et al. could not find any association between ARB intake and pneumonia development among the Taiwanese population [31]. Henry at al. support these findings with their results, which determined that ARB therapy did not affect the risk of being diagnosed with pneumonia within a hospital setting [32]. Interestingly, our study did find a slightly and not a relevant increased incidence of ALRI in patients under ARB therapy. However, since these findings were only marginally distinct, we do not consider them to be of clinical significance.

The third antihypertensive agent examined in our study—CCB—was associated with a slightly reduced ALRI incidence in women not being treated with antibiotics. Compared to ACEi and ARBs, CCB appears to be less frequently discussed regarding a possible association with ALRI manifestation. Therefore, little evidence is available to allow for an understanding of the possible impacts of CCB therapy on pulmonary inflammation. Furthermore, the few studies published remain contradictory in their findings: In their 2001 study, Arai and colleagues found no difference between cases and controls under CCB therapy regarding the risk of pneumonia [33]. By contrast, Mukamal et al. observed a modestly higher risk of pneumonia in patients under CCB therapy [13], which has been attributed to a possible suppression of lymphocyte activation [34]. To the best of our knowledge, we are among the first to discover a negative association between CCB intake and ALRI development in hypertensive patients. Our findings support the results of Lin et al., which outlined improved outcomes for respiratory insufficiency, bacteremia, and severe sepsis in patients under CCB therapy admitted to the hospital with pneumonia [35].

Although it can be stated in general that therapy with RAS-blocking antihypertensives was found to be associated with a higher risk of ALRI manifestation, the risk appears to be only slightly increased. Whether the evidence available today is biased by the fact that many studies are limited to Asian populations and other subpopulations remains open to speculation. Therefore, further studies are essential to understand the infection risks associated with antihypertensive therapy within a general population setting. Furthermore, the potential beneficial effect of CCB on pneumonia-related outcomes we identified within our analysis needs to be explored in future targeted studies.

Although this study is based on a large population of 377,470 patients and provides valuable insights concerning the potential infection risks associated with the intake of antihypertensive agents, it is subject to a number of weaknesses that should be considered: First, the database used did not include information on any socioeconomic factors, such as education, smoking status, alcohol consumption, and lifestyle. Second, data used in this analysis only include real-world data from an outpatient setting and do not include hospital data or data retrieved from specialists. Third, we only considered the monotherapeutic intake of antihypertensives and did not examine the combined application of antihypertensive agents. Finally, due to the retrospective nature of the analysis, no causal relationships could be reported; instead, we focused solely on associations. No clinical decisions on therapy strategies should be taken on the basis of associations alone.

## 5. Conclusions

In the present study, only slight and not clinically relevant increases or decreases in the ALRI incidence depending on antihypertensive therapy were observed. Additional studies are necessary to further explore the risks associated with antihypertensive agents widely embedded in today’s clinical practice.

## Figures and Tables

**Table 1:** Basic characteristics of the study sample after propensity score matching.

Variable	Proportion Affected among Patients Treated with ACE Inhibitors (%)	Proportion Affected among Patients Treated with Beta Blockers (%)	Proportion Affected among Patients Treated with Diuretics (%)	Proportion Affected among Patients Treated with CCB (%)	Proportion Affected among Patients Treated with ARB (%)	*p*-Value
N	75,494	75,494	75,494	75,494	75,494	
Age (Mean, SD)	65.3 (14.0)	65.3 (14.0)	65.3 (14.0)	65.3 (14.0)	65.3 (14.0)	1.000
Age ≤ 60	36.2	36.2	36.2	36.2	36.2	1.000
Age 61–70	23.3	23.3	23.3	23.3	23.3	
Age 71–80	26.4	26.4	26.4	26.4	26.4	
Age > 80	14.1	14.1	14.1	14.1	14.1	
Female	56.9	56.9	56.9	56.9	56.9	1.000
Male	43.1	43.1	43.1	43.1	43.1	
Diabetes	13.2	13.2	13.2	13.2	13.2	1.000
Ischemic heart diseases	6.6	6.6	6.6	6.6	6.6	1.000
Heart failure	1.7	1.7	1.7	1.7	1.7	1.000
Renal failure	2.0	2.0	2.0	2.0	2.0	1.000
COPD	3.7	3.7	3.7	3.7	3.7	1.000
Asthma	2.4	2.4	2.4	2.4	2.4	1.000

Proportions of patients given in % unless otherwise indicated. SD: standard deviation.

**Table 2:** Association between antihypertensive therapy (as compared to other antihypertensive therapy classes) and the incidence of pneumonia and acute bronchitis in patients followed in general practices in Germany (Cox regression models).

Cohort	BB versus Rest	CCB versus Rest	ARB versus Rest	DIU versus Rest	ACEI versus Rest
Total					
Diagnosis	0.96 (0.93–0.99)	0.93 (0.90–0.96)	1.08 (1.05–1.12)	1.02 (0.99–1.05)	1.02 (0.98–1.05)
Diagnosis + antibiotic prescription	0.95 (0.90–0.99)	0.96 (0.92–1.00)	1.09 (1.94–1.13)	1.03 (0.99–1.07)	0.99 (0.94–1.03)
Age ≤ 60					
Diagnosis	0.96 (0.91–1.02)	0.89 (0.85–0.95)	1.08 (1.03–1.14)	0.96 (0.91–1.02)	1.11 (1.05–1.17)
Diagnosis + antibiotic prescription	0.96 (0.89–1.03)	0.90 (0.84–0.96)	1.10 (1.03–1.17)	1.00 (0.94–1.07)	1.06 (0.99–1.13)
Age 61–70					
Diagnosis	1.01 (0.94–1.09)	0.97 (0.90–1.04)	1.06 (0.99–1.14)	0.94 (0.87–1.029	1.02 (0.95–1.10)
Diagnosis + antibiotic prescription	0.96 (0.87–1.06)	0.99 (0.90–1.09)	1.04 (0.95–1.14)	1.01 (0.91–1.10)	1.01 (0.92–1.11)
Age 71–80					
Diagnosis	0.95 (0.89–1.03)	0.93 (0.87–1.00)	1.12 (1.05–1.20)	1.06 (0.99–1.13)	0.94 (0.88–1.01)
Diagnosis + antibiotic prescription	0.98 (0.89–1.08)	0.96 (0.87–1.05)	1.14 (1.04–1.24)	1.01 (0.93–1.11)	0.93 (0.81–0.99)
Age > 80					
Diagnosis	0.92 (0.83–1.00)	0.94 (0.85–1.03)	1.08 (0.98–1.18)	1.10 (1.00–1.20)	0.99 (0.90–1.08)
Diagnosis + antibiotic prescription	0.88 (0.77–1.00)	1.09 (0.96–1.24)	1.08 (0.95–1.24)	1.02 (0.90–1.15)	0.95 (0.84–1.08)
Women					
Diagnosis	0.96 (0.92–1.00)	0.91 (0.87–0.95)	1.06 (1.02–1.11)	1.05 (1.01–1.10)	1.02 (0.98–1.06)
Diagnosis + antibiotic prescription	0.96 (0.91–1.02)	0.94 (0.89–1.00)	1.05 (0.99–1.11)	1.08 (1.02–1.14)	0.97 (0.91–1.02)
Men					
Diagnosis	0.95 (0.91–1.00)	0.94 (0.89–0.99)	1.12 (1.06–1.18)	0.98 (0.93–1.04)	1.02 (0.97–1.08)
Diagnosis + antibiotic prescription	0.91 (0.85–0.98)	0.96 (0.90–1.03)	1.14 (1.07–1.23)	0.97 (0.90–1.04)	1.02 (0.95–1.10)

**Table 3:** Association between antihypertensive therapy class (as compared to patients without therapy) and the incidence of pneumonia and acute bronchitis in patients followed in general practices in Germany (Cox regression models).

Cohort	BB versus No Therapy	CCB versus No Therapy	ARB versus No Therapy	DIU versus No Therapy	ACEI versus No Therapy
Total					
Diagnosis	0.95 (0.91–1.00)	0.98 (0.93–1.00)	1.10 (1.05–1.15)	1.06 (1.01–1.11)	1.05 (1.00–1.10)
Diagnosis + antibiotic prescription	0.95 (0.89–1.01)	1.03 (0.97–1.10)	1.12 (1.05–1.19)	1.08 (1.02–1.15)	1.02 (0.96–1.09)

## Data Availability

Data are however available from the corresponding author upon reasonable request.

## References

[1] Egan B.M., Kjeldsen S.E., Grassi G., Esler M., Mancia G. (2019). The global burden of hypertension exceeds 1.4 billion people. J. Hypertens..

[2] Mills K.T., Stefanescu A., He J. (2020). The global epidemiology of hypertension. Nat. Rev. Nephrol..

[3] Salem H., Hasan D.M., Eameash A., El-Mageed H.A., Hasan S., Ali R. (2018). Worldwide Prevalence of Hypertension: A Pooled Meta-Analysis of 1670 Studies In 71 Countries with 29.5 Million Participants. J. Am. Coll. Cardiol..

[4] Khalil H., Zeltser R. (2022). Antihypertensive Medications.

[5] Jackson R.E., Bellamy M.C. (2015). Antihypertensive drugs. Contin. Educ. Anaesth. Crit. Care Pain.

[6] Sarganas G., Knopf H., Grams D., Neuhauser H.K. (2016). Trends in Antihypertensive Medication Use and Blood Pressure Control Among Adults with Hypertension in Germany. Am. J. Hypertens.

[7] Vangen-Lønne A.M., Wilsgaard T., Johnsen S.H., Løchen M.L., Njølstad I., Mathiesen E.B. (2017). Declining Incidence of Ischemic Stroke: What Is the Impact of Changing Risk Factors? The Tromsø Study 1995 to 2012. Stroke.

[8] Pore P., Ghattargi C.H., Rayate M.R. (2010). Study of Risk Factors of Acute Respiratory Infection (ARI) in Underfives in Solapur. Natl. J. Community Med..

[9] Avendaño Carvajal L., Perret Pérez C. (2020). Epidemiology of Respiratory Infections. Pediatric Respiratory Diseases.

[10] Sekizawa K., Matsui T., Nakagawa T., Nakayama K., Sasaki H. (1998). ACE inhibitors and pneumonia. Lancet.

[11] Van de Garde E.M.W., Souverein P.C., Hak E., Deneer V.H.M., van den Bosch J.M.M., Leufkens H.G.M. (2007). Angiotensin-converting enzyme inhibitor use and protection against pneumonia in patients with diabetes. J. Hypertens.

[12] Lai C.C., Wang Y.H., Wang C.Y., Wang H.C., Yu C.J., Chen L. (2018). Comparative effects of angiotensin-converting enzyme inhibitors and angiotensin II receptor blockers on the risk of pneumonia and severe exacerbations in patients with COPD. Int. J. Chronic Obstr. Pulm. Dis..

[13] Mukamal K.J., Ghimire S., Pandey R., O’Meara E.S., Gautam S. (2010). Antihypertensive medications and risk of community-acquired pneumonia. J. Hypertens..

[14] Westendorp W.F., Vermeij J.D., Brouwer M.C., Roos Y.B.W.E.M., Nederkoorn P.J., van de Beek D. (2016). Pre-Stroke Use of Beta-Blockers Does Not Lower Post-Stroke Infection Rate: An Exploratory Analysis of the Preventive Antibiotics in Stroke Study. Cerebrovasc. Dis..

[15] Maier I.L., Becker J.C., Leyhe J.R., Schnieder M., Behme D., Psychogios M.N., Liman J. (2018). Influence of beta-blocker therapy on the risk of infections and death in patients at high risk for stroke induced immunodepression. PLoS ONE.

[16] Rathmann W., Bongaerts B., Carius H.J., Kruppert S., Kostev K. (2018). Basic characteristics and representativeness of the German Disease Analyzer database. Int. J. Clin. Pharm..

[17] Jacob L., Kostev K. (2018). Persistence with antihypertensive drugs in patients with depression in Germany. Int. J. Clin. Pharmacol. Ther..

[18] Warda A., Reese J.P., Tanislav C., Kostev K. (2019). The association between antihypertensive therapy and the incidence of Parkinson’s disease in patients followed in general practices in Germany. Int. J. Clin. Pharmacol. Ther..

[19] Tanislav C., Kostev K. (2022). Investigation of the prevalence of non-COVID-19 infectious diseases during the COVID-19 pandemic. Public Health.

[20] Kern W.V., Kostev K. (2021). Prevalence of and Factors Associated with Antibiotic Prescriptions in Patients with Acute Lower and Upper Respiratory Tract Infections—A Case-Control Study. Antibiotics.

[21] Kikuchi R., Watabe N., Konno T., Mishina N., Sekizawa K., Sasaki H. (1994). High incidence of silent aspiration in elderly patients with community-acquired pneumonia. Am. J. Respir. Crit. Care Med..

[22] Arai T., Yoshimi N., Fujiwara H., Sekizawa K. (2003). Serum substance P concentrations and silent aspiration in elderly patients with stroke. Neurology.

[23] Van de Garde E.M.W., Souverein P.C., van den Bosch J.M.M., Deneer V.H.M., Leufkens H.G.M. (2006). Angiotensin-converting enzyme inhibitor use and pneumonia risk in a general population. Eur. Respir. J..

[24] Lin S.Y., Chang S.S., Lin C.L., Lin C.C., Hsu W.H., Chou C.H., Chi C.Y., Lin C.D., Tu C.Y., Hsu C.Y. (2021). Association between angiotensin-converting enzyme inhibitors or angiotensin receptor blockers and community-acquired pneumonia: A nationwide population propensity-score matching study. Int. J. Clin. Pract..

[25] Caldeira D., Alarcão J., Vaz-Carneiro A., Costa J. (2012). Risk of pneumonia associated with use of angiotensin converting enzyme inhibitors and angiotensin receptor blockers: Systematic review and meta-analysis. BMJ.

[26] Liu C.L., Shau W.Y., Wu C.S., Lai M.S. (2012). Angiotensin-converting enzyme inhibitor/angiotensin II receptor blockers and pneumonia risk among stroke patients. J. Hypertens..

[27] Ohkubo T., Chapman N., Neal B., Woodward M., Omae T., Chalmers J. (2012). Effects of an Angiotensin-converting Enzyme Inhibitor-based Regimen on Pneumonia Risk. Am. J. Respir. Crit. Care Med..

[28] Dublin S., Walker R.L., Jackson M.L., Nelson J.C., Weiss N.S., Jackson L.A. (2012). Angiotensin-converting enzyme inhibitor use and pneumonia risk in community-dwelling older adults: Results from a population-based case-control study. Pharm. Drug Saf..

[29] Cao D.Y., Giani J.F., Veiras L.C., Bernstein E.A., Okwan-Duodu D., Ahmed F., Bresee C., Tourtellotte W.G., Karumanchi S.A., Bernstein K.E. (2021). An ACE inhibitor reduces bactericidal activity of human neutrophils in vitro and impairs mouse neutrophil activity in vivo. Sci. Transl. Med..

[30] Kim J., Lee J.K., Heo E.Y., Chung H.S., Kim D.K. (2016). The association of renin-angiotensin system blockades and pneumonia requiring admission in patients with COPD. Int. J. Chronic Obstr. Pulm. Dis..

[31] Liu C.L., Shau W.Y., Chang C.H., Wu C.S., Lai M.S. (2013). Pneumonia risk and use of angiotensin-converting enzyme inhibitors and angiotensin II receptor blockers. J. Epidemiol..

[32] Henry C., Zaizafoun M., Stock E., Ghamande S., Arroliga A.C., White H.D. (2018). Impact of angiotensin-converting enzyme inhibitors and statins on viral pneumonia. Bayl. Univ. Med. Cent. Proc..

[33] Arai T., Sekizawa K., Ohrui T., Fujiwara H., Yoshimi N., Matsuoka H., Sasaki H. (2005). ACE inhibitors and protection against pneumonia in elderly patients with stroke. Neurology.

[34] Nanni G., Panocchia N., Tacchino R., Foco M., Piccioni E., Castagneto M. (2000). Increased incidence of infection in verapamil-treated kidney transplant recipients. Transpl. Proc..

[35] Zheng L., Hunter K., Gaughan J., Poddar S. (2017). Preadmission Use of Calcium Channel Blockers and Outcomes After Hospitalization with Pneumonia: A Retrospective Propensity-Matched Cohort Study. Am. J. Ther..

